# MicroRNAs in the epigenetic regulation of disease progression in Parkinson’s disease

**DOI:** 10.3389/fncel.2022.995997

**Published:** 2022-09-16

**Authors:** Sushmaa Chandralekha Selvakumar, K. Auxzilia Preethi, Deusdedit Tusubira, Durairaj Sekar

**Affiliations:** ^1^Centre for Cellular and Molecular Research, Saveetha Dental College and Hospitals, Saveetha Institute of Medical and Technical Sciences, Saveetha University, Chennai, India; ^2^Department of Biochemistry, Mbarara University of Science and Technology, Mbarara, Uganda

**Keywords:** Parkinson’s disease, microRNA, epigenetic regulation, neurons, signaling pathways

## Abstract

Parkinson’s disease (PD) is a multifactorial neurodegenerative condition with symptoms such as resting tremor, rigidity, bradykinesia (slowness of moment), and postural instability. Neuroinflammation plays a significant part in the onset and progression of neurodegeneration in a wide range of disorders, including PD. The loss of dopaminergic neurons in the substantia nigra (SN) is thought to be the primary cause of PD disease progression. However, other neurotransmitter systems like serotoninergic, glutamatergic, noradrenergic, adrenergic, cholinergic, tryptaminergic, and peptidergic appear to be affected as well. Epigenetic regulation of gene expression is emerging as an influencing factor in the pathophysiology of PD. In recent years, epigenetic regulation by microRNAs (miRNAs) has been discovered to play an important function in the disease progression of PD. This review explores the role of miRNAs and their signaling pathways in regulating gene expression from development through neurodegeneration and how these mechanisms are linked to the pathophysiology of PD, emphasizing potential therapeutic interventions.

## Introduction

Parkinson’s disease (PD) is the second most common neurodegenerative disorder, affecting 1–2 people per 1,000 at any moment. The prevalence of PD rises with age, and it affects 1% of the population over the age of 60 (Tysnes and Storstein, 2017; Ou et al., 2021). The origin of PD is still unknown, and it’s postulated to be caused by the lack of dopamine due to the loss or degeneration of dopaminergic neurons in the substantia nigra par compacta (SNpc), a part of the brain that controls movement. Since dopamine acts as an inhibitor in the basal ganglia, it limits extrapyramidal movement (Ou et al., 2021). The pathophysiology of PD involves the accumulation of neuronal Lewy Bodies, and the risk factors include aging, family history, pesticide exposure, and environmental pollutants. Bradykinesia (slowness of movement), resting tremor (involuntary twitching or shaking of muscles), rigidity (involuntary stiffening of muscles), postural instability (inability to maintain equilibrium), hyposmia (decreased sense of smell) and rapid eye moment are all effects of PD (Beitz, 2014).

Since the symptoms of PD involve motor functions, most of the drugs used in the treatment of PD are dopamine based. Commercially available dopamine agonists exhibit significant improvement in PD, yet they are also susceptible to many adverse effects. This makes it challenging to use in many patients. The pharmacological drugs lack specificity, leading to severe side effects. All these limitations are due to inadequate knowledge of the molecular mechanisms, signaling pathways and epigenetic regulations involved in the disease progression of PD. Diagnosis of PD is possible only after the onset of symptoms, and there is a lack of validated biomarkers for early diagnosis of PD (Rizek et al., 2016; Armstrong and Okun, 2020). Research on biomarkers could help in early diagnosis as well as could pave for early treatment of PD.

Interestingly, in recent years, many studies have focused on the molecular mechanisms and epigenetic regulation of disease progression in PD. Yet there are no definitive results due to the complexity of the disease. There are specific molecules that are involved in the regulation of various signaling pathways and genes. One such important regulator is microRNA (miRNAs) which are small non-coding RNAs found to be involved in the expression and suppression of various genes.

This review paper focuses on miRNAs involved in the molecular mechanisms, signaling pathways and epigenetic regulations in the disease progression of PD.

## MicroRNAs and their biogenesis

**FIGURE 1:**
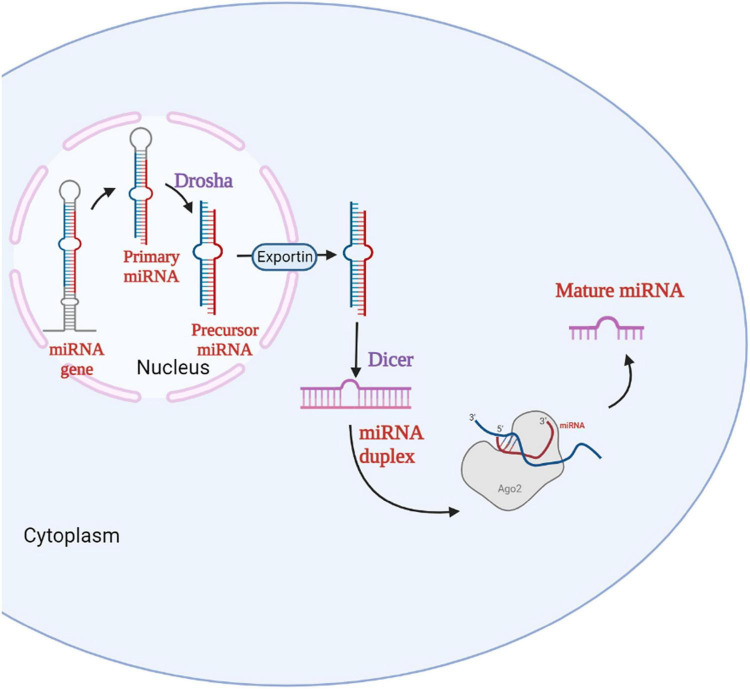
Biogenesis of miRNA. The miRNA gene in the nucleus is transcribed to primary miRNA which is then converted to precursor miRNA by the enzyme Drosha. The precursor miRNA enters the cytoplasm via Exportin and forms the miRNA duplex with the help of Dicer. The miRNA duplex forms the mature miRNA with the help of RNA-Induced Silencing Complex (RISC).

miRNAs are short non-coding RNA molecules with approximately 17–22 nucleotides in length that controls gene expression post-transcriptionally through either translational repression or mRNA degradation (Vishnoi and Rani, 2017). miRNAs have a significant role in the regulation of cell proliferation, differentiation, and apoptosis in various cellular processes. The biogenesis of miRNAs begins with the processing of RNA polymerase II/III. miRNA biogenesis is divided into two pathways, namely canonical and non-canonical pathways (O’Brien et al., 2018). The canonical biogenesis pathway is the most common approach for miRNA processing. In the canonical pathway, at first, the primary miRNA (pri-miRNA) is transcribed from their gene and processed into precursor miRNA (pre-miRNA) by the microprocessor complex, which consists of an RNA binding protein called DiGeorge Syndrome Critical Region 8 (DGCR8) and a ribonuclease III enzyme called Drosha (Saliminejad et al., 2019) which takes place in the nucleus. Once pre-miRNAs are synthesized, an exportin 5 (XPO5)/RanGTP complex transports them to the cytoplasm, where the second processing step occurs, where they are processed by the RNase III endonuclease called Dicer (Denli et al., 2004). The terminal loop is removed during this step, which results in the formation of the mature miRNA duplex. The mature miRNA form is named according to the miRNA strand’s directionality (Bartel, 2004). The 5p strand emerges from the pre-miRNA hairpin’s 5’ end, while the 3p strand emerges from the 3’ end. The argonaute (AGO) family proteins play a crucial function in RNA silencing. With the help of ATP- dependent chaperone proteins, the miRNA duplex is loaded into the AGO protein (Vishnoi and Rani, 2017). The passenger strand of the miRNA duplex is released once the AGO is returned to its regular conformation, resulting in single-stranded mature miRNA (Saliminejad et al., 2019). After loading, AGO stimulates the formation of a ribonucleoprotein complex known as RNA-induced silencing complex (RISC), which facilitates the recognition of the targeted mRNA (Ha and Kim, 2014). Figure 1 represents the miRNA biogenesis in the canonical pathway.

Apart from the above-mentioned canonical miRNA biogenesis processes, a number of additional methods can produce miRNAs: one such pathway is the non-canonical miRNA biogenesis pathway. Non-canonical miRNA biogenesis can be further divided into two categories, namely Drosha/DGCR8-independent and Dicer-independent pathways (Abdelfattah et al., 2014). The Drosha/DGCR8-independent process produces pre-miRNAs that look similar to that of Dicer substrates. Mirtrons, formed from the introns of mRNA during splicing, are an example of pre-miRNAs (Havens et al., 2012). Spliceosomes and debranching enzymes process mirtrons to produce acceptable dicer substrates, bypassing the microprocessor stage (Havens et al., 2012). On the other hand, Drosha processes Dicer-independent miRNAs from endogenous short hairpin RNA (shRNA) transcripts (Chong et al., 2010). However, recent research on simtrons and other Dicer-independent miRNAs has uncovered new, non-canonical pathways that are not only unexplored but also crucial in understanding the miRNA synthesis without Dicer. Overall, the findings suggest that Dicer, which is known to be important in both canonical and non-canonical pathways, is not required for some miRNAs that have yet to be investigated.

At times, these miRNAs are encapsulated within the exosomes, which are extracellular vesicles and are termed exosomal miRNAs. Since the exosomes are distributed in the body fluids like blood, saliva, cerebrospinal fluid (CSF) and urine, the exosomal miRNAs are identified to be important liquid biopsy biomarkers. Due to the blood-brain barrier system, it is difficult to identify the miRNAs from the central nervous system (CNF) in the blood. Hence, differential expression of miRNAs could be found in the CSF of neurodegenerative patients. Since the exosomes act as cargo, the exosomal miRNAs travel from the CNS to the peripheral nervous system (PNS) and affect the motor functions in the patients by targeting the important genes in PD (Rastogi et al., 2021). Moreover, since the exosomes can incorporate into the cells through receptor-ligand interaction, it makes it easier for the miRNAs to spread and reach the target genes (Zhang et al., 2019).

## MicroRNAs involved in signaling mechanisms and epigenetic regulation of Parkinson’s disease

Research on the role of miRNAs in the pathophysiology of PD is vast, and various miRNAs are involved in the regulation of signaling pathways in the disease progression of PD. For instance, miRNAs like miR-133b and miR- 384-5p are found to be regulating apoptosis in PD (Dong et al., 2020; Tao et al., 2020). Likewise, the following miRNAs mediate different signaling pathways and epigenetic regulation in PD. Table 1 represents the miRNA involved in the signaling pathways of Parkinson’s disease.

**TABLE 1:** miRNAs involved in the signaling pathways of Parkinson’s disease.

S. no.	MicroRNA	Target gene	Function	References
1	miR-133b	ERK 1/2	Apoptosis	Dong et al., 2020
2	miR-384-5p	SIRT 1	Apoptosis	Tao et al., 2020
3	miR-124	p62, p38	Inflammatory response	Yao et al., 2019
4	Let-7b-3p	HMGA2	Cell apoptosis and inflammation	Huang et al., 2021
5	miR-29c-3p	NFAT5	Inflammation and Autophagy	Wang R. et al., 2021
6	miR-103a-3p	Parkin	Mitophagy	Zhou et al., 2021
7	miR-96	BNIP3	Mitophagy	Wang D. X. et al., 2021
8	miR-17	DNMT1	DNA methylation	Zhang et al., 2021
9	miR-335	FTH1	Ferroptosis	Li et al., 2021
10	miR-29c-3p	TET2	Autophagy	Wang R. et al., 2021
11	miR-326	XBP1	Autophagy	Zhao et al., 2019
12	miR-15b-5p	GSK-3	Autophagy and Cell death	Zhu J. et al., 2021
13	miR-216a	Bax	Cell death	Yang et al., 2020
14	miR-421	MEF2D	Neurotoxicity and autophagy	Dong et al., 2021
15	miR-425	–	Dopaminergic activity	Hu et al., 2019
16	miR-497-5p	FGF2	Autophagy	Zhu W. et al., 2021
17	miR-3473b	TREM2/ULK1	Autophagy and Inflammation	Lv et al., 2021

### MicroRNAs and inflammation mediating pathways in Parkinson’s disease

In a study by Yao et al. (2019), it was identified that microglial activation is regulated by miR-124’s aberrant expression. Their results suggested miR-124 as a potential therapeutic target for controlling the inflammatory response in PD. It was concluded that by targeting p62, p38, and autophagy, miR-124 might be able to reduce neuroinflammation during the development of PD (Yao et al., 2019). Moreover, high amounts of let-7b-5p were discovered in the brain tissues of PD animals and MPP + -treated SH-SY5Y cells. Let-7b-5p knockdown prevented SH-SY5Y cell death. The study found that let-7b-5p contributes to cell apoptosis in PD via targeting HMGA2, thus providing a therapeutic target for PD (Huang et al., 2021). In one of the studies, miR-29b2/c was found to be involved in aging and disease progression in PD. Using miR-29b2/c gene knockout mice, the role of miR-29b2/c in aging and PD was investigated. Lack of miR-29b2/c increased AMPK activation while suppressing NF-κB/p65 signaling in glial cells. Thus, the study concluded the detrimental effect miR-29b2/c had on PD (Bai et al., 2021).

### MicroRNAs and mitophagy mediating pathways in Parkinson’s disease

In PD, the effects of mitophagy mediated by miR-103a-3p/Parkin/Ambra1 signaling were investigated. Parkin expression was reduced by miR-103a-3p, and miR-103a-3p inhibition had neuroprotective benefits in Parkinson’s disease (PD). This suggests that miR-103a-3p is involved in controlling mitophagy via the Parkin/Ambra1 pathway (Zhou et al., 2020). Interestingly, a study compared two miRNAs, miR-17 and miR-19a in the regulation of DNA methyltransferases (DNMT) in PD. It was identified that miR-17 regulated DNMT1 and was responsible for abnormal DNA methylation occurring in PD. Thus miR-17 was identified to be a therapeutic modulator of DNA methylation in PD (Zhang et al., 2021). Interestingly, miR-96 and pramipexole (PPX) which plays a protective role in PD was analyzed in PD cell line (1-methyl 4-phenylpyridinium (MPP^+^) induced cells) and 1-methyl-4-phenyl-1,2,3,6-tetrahydropyridine (MPTP) induced mice. It was identified that to prevent PTEN-induced putative kinase 1 (PINK1)/Parkin signals from regulating mitophagy, miR-96 specifically targeted BNIP3. By reducing BNIP3, miR-96 overexpression facilitated MPP + -induced neuronal damage. By controlling miR-96/BNIP3-mediated mitophagy, PPX reduced MPTP-induced neuronal damage in mice. Thus miR-96 was found to be playing an important role in PD (Wang D. X. et al., 2021).

### MicroRNAs and ferroptosis mediating pathways in Parkinson’s disease

Fascinatingly, the process of iron (Fe^2+^)-dependent programmed cell death is known as ferroptosis. Ferroptosis is strongly related to the pathogenic alterations seen in PD, including nigral iron elevation, glutathione depletion, and enhanced reactive oxygen species (ROS) generation. miR-335 was found to enhance ferroptosis by degrading FTH1. The results of the study showed that miR-335 promotes ferroptosis in both *in vitro* and *in vivo* models of PD by targeting FTH1 (Li et al., 2021). Moreover, miR-221 controls PC12 cell viability and apoptosis by targeting PTEN, providing protection against PD. Consequently, miR-221 may be a viable therapeutic target for the treatment of PD (Li L. et al., 2018).

### MicroRNAs and autophagy mediating pathways in Parkinson’s disease

Autophagy in dopamine neurons is thought to be linked to Parkinson’s disease, but the precise mechanism is uncertain. In one of the studies, the findings showed that autophagy was induced in the SNpc dopaminergic neurons of PD animals due to the downregulation of miR-29c-3p and upregulation of 10–11 translocation 2 (TET2) expression. In PD animals, up-regulation of miR-29c-3p reduced TET2 expression and SNpc autophagy, which includes dopaminergic neurons. Overall, over-expression of miR-29c-3p in PD models reduces autophagy, which may be caused by TET2. The findings exhibited the role of miR-29c-3p in controlling autophagy to accelerate the progression of PD (Wang R. et al., 2021). One of the studies sought to understand the targeting of X-box binding protein 1 (XBP1) by miR-326 in the biological functions of PD. Mice treated with a miR-326 mimic and siRNA-XBP1 displayed improved traction test results, autophagy activation, LC3-II, c-Jun, and p-Syn expression, but decreased climbing time and iNOS, α-Syn, and p-c-Jun expression. According to the study’s findings, overexpression of miR-326, which specifically targets XBP1, reduces iNOS production and encourages autophagy of dopaminergic neurons through JNK signaling (Zhao et al., 2019).

One study examined how inhibiting miR-15b-5p will suppress cell death in Parkinson’s disease (PD) by focusing on the Akt3-mediated GSK-3/catenin signaling pathway (Zhu J. et al., 2021). Another study found that miR-216a controlled the development of PD through controlling Bax, suggesting that miR-216a could be a possible target for PD (Yang et al., 2020). It was discovered that increased miR-421 encourages cell death by inhibiting MEF2D expression. In cellular and animal models of PD, miR-421 inhibition or MEF2D restoration protected neurons from neurotoxicity (Dong et al., 2021). According to a study, mice treated with MPTP had their miR-425 levels increased, which prevented the defective degradation of dopaminergic neurons and improved behavioral impairments. These results point to miR-425 brain administration as a possible therapeutic strategy for the treatment of Parkinson’s disease (Hu et al., 2019). Interestingly, another study demonstrated that blocking miR-497-5p caused PD mice’s bradykinesia to improve, cell apoptosis to be reduced, and autophagy to be activated by FGF2. As a result of inhibiting cell apoptosis and boosting autophagy in a FGF2 dependent manner, miR-497-5p silencing alleviates PD and offers a fresh target for treating PD (Zhu W. et al., 2021).

In addition, the role of TREM2 and ULK1 in PD were analyzed. It was discovered that up-regulating TREM2 greatly boosted the expression of p-ULK1. Increased p-ULK1 expression had the ability to suppress inflammatory factor expression while promoting autophagy. Through TREM2, miR-3473b may indirectly control ULK1. In order to control the involvement of autophagy in the pathogenesis of inflammation in PD, miR-3473b may affect TREM2/ULK1 expression, which suggests that it may be a possible therapeutic target to control the inflammatory response in PD (Lv et al., 2021).

## MicroRNAs as biomarkers and therapeutic targets in Parkinson’s disease

miRNAs are being studied for their potential role as biomarkers and therapeutic targets in several diseases including neurodegenerative disorders. Once the molecular mechanisms and signaling pathways are elucidated, their role as biomarkers and therapeutic targets could be easily explained.

One of the crucial components for the preservation of dopaminergic function and one that is particularly sensitive in PD is nuclear receptor related 1 protein (Nurr1), which is targeted by miR-132 and their expression is adversely linked (PD). Thus miR-132 could serve as a biomarker as well as potential therapeutic target (Yang et al., 2019). In one of the studies, the CSF of PD patients were collected and differential expression of miR-626 was analyzed. The results revealed that miR-626 was significantly downregulated in PD patients when compared to normal controls (Qin L. X. et al., 2021). Engrossingly, the serum miR-150 levels in PD patients was found to be differentially expressed. The miR-150 levels in the serum of PD patients were less compared to healthy normal. It was also identified that miR-150 induced inflammation by targeting the AKT3 gene (Li et al., 2020).

Another study by Han et al. (2020) showed that miR-29 levels in the serum are related to cognitive dysfunction in PD. It has been demonstrated that miR-29b levels are connected with several subtypes of PD cognition and may successfully distinguish PD associated dementia (PDD) from non-PDD (Han et al., 2020). A study by Qin X. et al. (2021) explored the role of miR-185 in PD. It was identified that miR-185 levels were decreased in PD-affected rats. In the substantia nigra of PD rats, miR-185 restoration improved pathological damage, oxidative stress, and inhibited neuronal death. To stimulate the PI3K/AKT signaling pathway, miR-185 targeted IGF1. IGF1 upregulation reduced the effects of restored miR-185 on PD animals. Thus, the study concluded miR-185 as a potential therapeutic target for PD (Qin X. et al., 2021).

Through the control of LIM and SH3 domain protein 1 (LASP1), one study investigated the protective effect of miR-218-5p on dopaminergic neuron damage in SNpc of rats with PD. It was confirmed that miR-218-5p targets LASP1. In the brain SNpc of PD rats, increased miR-218-5p or decreased LASP1 reduced dopaminergic neurons’ oxidative stress and death. Furthermore, elevated miR-218-5p suppressed LASP1 expression in the SNpc of the brain of PD rats. According to the study, up-regulated miR-218-5p may help PD rats’ damaged dopaminergic neurons (Ma et al., 2021). In PD animal and neuronal models, one study found that miR-29c-3p (miR-29c) had anti-inflammatory characteristics. The release of pro-inflammatory cytokines as well as the activation of the NF-êB and TXNIP/NLRP3 inflammasome were inhibited by miR-29c overexpression. Additionally, it was discovered that miR-29c and NFAT5 had a negative correlation. In microglia treated with miR-29c inhibitor, NFAT5 knockdown prevented the inflammation from becoming more severe. Therefore, these results imply that miR-29c targets NFAT5, which is a prospective therapeutic target for PD, and controls the NLRP3 inflammasome to inhibit microglial inflammatory responses (Wang et al., 2020).

Thus, the miRNAs played an important role in the signaling pathways involved in PD and they could be used as potential biomarker as well as therapeutic target. Table 2 represents the miRNAs as biomarkers and therapeutic targets in Parkinson’s disease.

**TABLE 2:** miRNAs as biomarkers and therapeutic targets in Parkinson’s disease.

S. no.	MicroRNA	Target gene	Function	References
1	miR-132	Nurr1	Biomarker and therapeutic target	Yang et al., 2019
2	miR-626	–	Biomarker	Qin X. et al., 2021
3	miR-150	AKT3	Biomarker	Li et al., 2020
4	miR-29b	–	Biomarker	Han et al., 2020
5	miR-185	IGF1	Therapeutic target	Qin X. et al., 2021
6	miR-218-5p	LASP1	Therapeutic target	Ma et al., 2021
7	miR-29c	NFAT5	Therapeutic target	Wang et al., 2020

## MicroRNAs in the pathophysiology of Parkinson’s disease involving genes

miRNAs are found to be involved in the disease pathogenesis of PD by regulating the various genes which play a major role in PD. Following are certain miRNAs regulating important genes involved in PD. Table 3 represents the miRNA regulating specific genes in Parkinson’s disease.

**TABLE 3:** miRNA regulating specific genes in Parkinson’s disease.

S. no.	Gene	miRNA	Function	References
1	Brain-Derived Neurotrophic Factor (BDNF)	miR-210-3p	Dopamine neuron survival	Zhang et al., 2018
2		miR-30e	BDNF secretion in SNpc	Li et al., 2019
3		miR-7	BDNF/α-Synuclein	Li et al., 2019
4		miR-494-3p	Neurotoxicity	Deng et al., 2020
5	Leucine-rich repeat kinase 2 (LRRK2)	miR-146a miR-335-3p miR-335-5p	LRRK2 mutation	Oliveira et al., 2020
6		miR-4671-3p, hsa-miR-335-3p, hsa-miR-561-3p, hsa-miR-579-3p, and hsa-miR-3143	LRRK2 mutation	Yılmaz et al., 2016
7		miR-335	Chronic neuroinflammation	Oliveira et al., 2021
8		miR-599	Brain cells protection	Wu Q. et al., 2019
9		miR-205	LRRK2	Zhou et al., 2021
10		miR-712	Inflammation	Talari et al., 2017
11		miR-7	LRRK2 mutation	Huang et al., 2022
12		miR-30c-5p	LRRK2 mutation	Yousefi et al., 2022
13	Death-Associated Protein Kinase 1 (DAPK1)	miR-26a	Loss of dopaminergic neurons	Su et al., 2019
14		miR-151-3p	Neuroprotection	Guo et al., 2021
15	Glutamate Transporter (GLT1)	miR-30a-5p	Glutamate Excitotoxicity	Meng et al., 2021
16		miR-543-3p	Dyskinesia	Wu X. et al., 2019
17	Tumor Growth factor-β (TGF-β)	miR-221-3p	Dopaminergic inflammation	Lang et al., 2022

### Brain-derived neurotrophic factor

The growth factor known as Brain-Derived Neurotrophic Factor (BDNF) is a member of the neurotrophin family and is essential for the survival of existing neurons as well as the promotion of neurogenesis. The loss of BDNF in the substantia nigra pars compacta (SNpc) causes a dopaminergic deficit in the striatum, which is a key component of PD (Eyileten et al., 2021). A study discussed that 1-Methyl-4-phenylpyridinium (MPP +) controls the production of BDNF through miR-210-3p. Through a transcription-unrelated mechanism, MPP + prevents the synthesis of BDNF in SH-SY5Y cells. Additionally, MPP + was found to increase the expression of miR-210-3p, which targets the BDNF mRNA in SH-SY5Y cells. Moreover, miR-210-3p suppression increases DA neuron survival in the MPTP animal model and prevents MPP + from reducing BDNF synthesis (Zhang et al., 2018).

In a study by Li D. et al. (2018), it was identified that by decreasing NLRP3 inflammasome activation and negatively regulating Nlrp3 expression in the MPTP-induced PD mouse model, miR-30e reduces neuronal injury, neuroinflammation, and dyskinesia. In this work, it was discovered that miR-30e agomir administration significantly restored the decrease in BDNF secretion in SNpc. A prolonged decline in BDNF mRNA expression can be seen in SNpc of PD patients, and abnormal changes in BDNF expression or signaling may play a role in neurodegeneration (Li D. et al., 2018). In addition, through an auto-regulatory mechanism, miR-7 controls the expression of BDNF and causes the downregulation of α-synuclein (α-syn), which is associated with the neuropathology of PD. These results suggest that miRNA-7 modulates the BDNF/α-syn axis in the early stages of Parkinson’s disease and may be used as a biomarker or therapeutic target (Li et al., 2019).

Interestingly, miR-494-3p was found to be upregulated in PD and was found to be targeting BDNF leading to neurotoxicity. Thus, inhibition of miR-494-3p was found to reverse the effect and increase BDNF levels. Hence, miR-494-3p is found to be a potential target for PD (Deng et al., 2020).

Moreover, BDNF was clinically tested for Alzheimer’s disease and an adenovirus vector (AAV2) was used as a carrier. Since in animal models, BDNF was found to reduce cell loss, promote cell function, and create fresh connections (synapses) between brain cells, it was subjected to phase I clinical trials. The study is currently recruiting candidates for the trials.^1^ Thus it could be understood that BDNF is an important gene that plays an important role in neurodegenerative disorders including PD.

### Leucine-rich repeat kinase 2

Leucine-rich repeat kinase 2 (LRRK2) is a key player in the genesis of PD. Mutations in LRRK2 are the main cause of both inherited and spontaneous PD. One of the research examined 45 people who had the LRRK2 gene mutated (LRRK2-PD). It was discovered that miR-155 was upregulated in LRRK-PD and that miR-146a, miR-335-3p, and miR-335-5p were downregulated in LRRK2-PD. Thus, these miRNAs were found to be involved in the mutation of the LRRK2 gene in PD patients (Oliveira et al., 2020). Interestingly, in a study by Yılmaz et al. (2016), hsa-miR-4671-3p, hsa-miR-335-3p, hsa-miR-561-3p, hsa-miR-579-3p, and hsa-miR-3143 were found to be differentially expressed in the whole blood of PD patients and these miRNAs were found to be targeting LRRK2 gene. Among these miRNAs, has-miR-561-3p was found to be the highly specific miRNA targeting LRRK2 in PD (Yılmaz et al., 2016).

Moreover, in various PD-mimicking circumstances, miR-335 is markedly downregulated, and miR-335 specifically targeted LRRK2 mRNA. The expression of pro-inflammatory genes induced by α-synuclein was reduced by miR-335. The effects of conventional inflammatory stimuli or LRRK2-Wt overexpression are greatly inhibited by miR-335, which, in turn, attenuates chronic neuroinflammation in both microglia and neuronal cells (Oliveira et al., 2021). Another study discovered that miR-599 protected brain cells by controlling the expression of LRRK2. In the brain tissues of PD animals, LRRK2 was substantially expressed compared to miR-599’s low expression. Additionally, LRRK2 expression at the mRNA and protein levels could be negatively regulated by miR-599. By suppressing the expression of LRRK2, miR-599 overexpression prevented SHP-SY5Y cells from being damaged by MPP + treatment (Wu Q. et al., 2019). Thus miR-599 was identified as a potential therapeutic target for PD.

In fact, it was discovered that the conserved binding site at the 3’-untranslated region (UTR) of the LRRK2 gene allowed miR-205 to inhibit the expression of the LRRK2 protein. It is interesting to note that patients with sporadic PD had much lower levels of miR-205 expression in their brains, which was indicative of elevated LRRK2 protein levels. Studies conducted *in vitro* on primary neuron cultures and cell lines further confirmed the function of miR-205 in regulating the production of the LRRK2 protein. In addition, the injection of miR-205 corrected the neurite outgrowth abnormalities in the neurons expressing a PD-related LRRK2 mutant. Collectively, these results imply that miR-205 may be downregulated, which may contribute to the potential pathogenic elevation of LRRK2 protein in the brains of sporadic PD patients, while miR-205 overexpression may offer a useful therapeutic approach to suppress the abnormal upregulation of LRRK2 protein in PD (Cho et al., 2013). Similarly, miR-199a-3p was found to be involved in the regulation of LRRK2 in the disease pathogenesis of PD (Zhou et al., 2021).

In addition, it was discovered that miR-712 targets the robust inflammatory gene LRRK2 and inhibits p38 and ERK1/2 kinase activation (Talari et al., 2017). However, their role in PD is yet to be elucidated. Another study by Huang et al., 2022 exhibited that LRRK2 degradation was accelerated by NEDD4 which is targeted by the miR-7/STAT3 axis. Thus, the study proposed the mechanisms of bone marrow mesenchymal stem cells derived exosomal TSG-6 to be a regulator of the STAT3/miR-7/NEDD4/LRRK2 axis (Huang et al., 2022). Additionally, miR-30c-5p was identified to target LRRK2 and play an important role in the pathogenesis of PD. It was also postulated that the long non-coding RNA Linc00938 could bind directly to hsa-miR-30c-5p, perhaps influencing LRRK2 expression via the miR-30c-5p sponge (Yousefi et al., 2022).

### Death-associated protein kinase 1

A common serine/threonine (Ser/Thr) kinase known as death-associated protein kinase 1 (DAPK1) is essential for cell death in a number of neurological diseases. One of the research found a favorable correlation between neuronal synucleinopathy and DAPK1 overexpression in PD animals. Additionally, it was discovered that synucleinopathy, DA neuron cell death, and motor impairments arise from downregulating miR-26a or upregulating DAPK1. By directly phosphorylating α-synuclein at Ser129, DAPK1 overexpression encourages PD-like symptoms. In line with this, motor problems, synucleiopathy, and the loss of dopaminergic neurons were all prevented by a cell-permeable competitive peptide that prevents the phosphorylation of α -synuclein (Su et al., 2019). miR-26a is found to be influencing DAPK in PD and hence it could be used as a potential therapeutic target for the treatment of PD. Likewise, miR-151-3p is also found to be increasing the neuroprotection by targeting DAPK (Guo et al., 2021).

### Glutamate transporter 1

PD can be characterized by glutamate excitotoxicity, which is brought on by malfunctioning GLT1. However, the processes behind the control of GLT in PD are still not completely understood. One of the studies demonstrated that mice treated with MPTP and astrocytes treated with MPP+, miR-30a-5p reduced GLT-1 expression and function. It was also discovered that miR-30a-5p knockdown boosted GLT-1 expression and accelerated glutamate absorption both *in vitro* and *in vivo* by preventing GLT-1 ubiquitination and subsequent degradation in a PKCα-dependent way (Meng et al., 2021). Another investigation discovered that in mice treated with MPTP and astrocytes treated with MPP+, miR-543-3p can inhibit GLT-1 expression and function. Suppression of miR-543-3p can alleviate dyskinesia and restore GLT-1 expression and function in the PD model, which raises the possibility that miR-543-3p inhibition could be used as a possible therapeutic target for PD (Wu X. et al., 2019). The studies summarized the role of miRNAs in GLT1 dysfunction which is identified to be playing important role in the disease progression of PD.

### Tumor growth factor-β1

A pleiotropic cytokine with immunosuppressive and anti-inflammatory effects is TGF-β1. Recent research has demonstrated that pretreatment with TGF-β1 *in vitro* prevents the death of dopaminergic neurons caused by MPP+, which is a hallmark of PD (Chen et al., 2017). Thus TGF-β1 is identified to be an important gene involved in dopaminergic inflammation which could be regulated by several miRNAs. But studies are sparse on the role of miRNAs in regulating TGF-β1 in PD. Interestingly, a study by Lang et al. (2022) investigated the interactions between LncRNA MIAT and miR-221-3p regulated TGF-β1/Nrf2. The study employed the use of a miR-221-3p mimic, a miR-221-3p inhibitor, an NC-inhibitor, and a shRNA (shTGF-β1) which were transfected into MPP+-treated cells. To examine the interaction of miR-221-3p with MIAT or TGFB receptor 1 (TGFBR1), dual-luciferase reporter gene experiments were done. As a consequence, LncRNA MIAT was abundantly produced in PD animals and cells, but LncRNA MIAT downregulation enhanced neuron survival, prevented apoptosis, and reduced oxidative stress in neurons. LncRNA MIAT is bound to miR-221-3p, and the expression of LncRNA MIAT was negatively correlated with miR-221-3p. Furthermore, miR-221-3p inhibited TGF-β1 expression while increasing Nrf2 expression. MIAT, a LncRNA, enhanced MPP+-induced neuronal damage in PD through modulating the TGF-β1/Nrf2 axis by interaction with miR-221-3p (Lang et al., 2022).

## Future prospects

One of the most widely expressed miRs in the brain, miR-124 is involved in autophagy, inflammation, synapse architecture, neurotransmission, and neurogenesis. miR-124 is found to be acting through the pathways of calpain 1/p25/cyclin-dependent kinases 5 (CDK5), signal transducer and activator of transcription 3 (STAT3) and Bcl-2-interacting mediator of cell death (Bim). Moreover, there is also evidence of miR-124 regulating nuclear factor-kappa B (NF-κB), AMPK and ERK signaling (Angelopoulou et al., 2019). Thus miR-124 being an important therapeutic target makes it a potential treatment strategy for PD. There are also studies on miR-34a whose reduction or inhibition increased neuronal survival against numerous neurotoxins linked to PD. miR-34a is found to be regulating various signaling pathways like Nrf2, SIRT1/mTOR and Notch signaling in the pathophysiology of PD (Ba et al., 2015; Kou et al., 2016; Chua and Tang, 2019). miR-34a not only regulates these signaling pathways but also plays a major role in the disease progression in PD.

Since various miRNAs are being studied for their potential role in regulating various genes and signaling pathways, they also could be used as potential therapeutic targets and biomarkers for PD. miRNA-based therapeutics have been researched on wide-scale nowadays due to their potential therapeutic properties. But the research on miRNA mimics and miRNA inhibitors in PD is sparse. Hence there is a requirement for many validated studies to seek a clinical breakthrough in miRNA-based therapeutics in PD.

Moreover, research on biomarkers could be useful in identifying various blood markers that help in the early diagnosis and monitoring of the disease. Since there is a need for early diagnosis, research on miRNAs could pave way for the prognosis and diagnosis of PD.

## Conclusion

Parkinson’s disease is a commonly found neurodegenerative disease with late-onset symptoms making it difficult to diagnose at earlier stages. Moreover, the pharmacological treatment for PD currently lacks specificity and has adverse effects. Hence there is a need for studies on biomarkers and therapeutic targets. miRNAs being suitable candidates are being researched for their role in disease progression in PD. miRNAs are found to be regulating various genes and their signaling pathways in PD. Hence, they could be used as biomarkers as well as therapeutic targets.

## Author contributions

DS and DT conceived the idea. DS wrote the first draft of the manuscript. SS, KP, DT, and DS wrote the complete manuscript. All authors contributed to the article and approved the submitted version.

## Footnotes

1
https://www.clinicaltrials.gov/ct2/show/NCT05040217
